# Blackcurrant (*Ribes nigrum* L.) Extract Exerts Potential Vasculoprotective Effects in Ovariectomized Rats, Including Prevention of Elastin Degradation and Pathological Vascular Remodeling

**DOI:** 10.3390/nu13020560

**Published:** 2021-02-08

**Authors:** Kayo Horie, Naoki Nanashima, Hayato Maeda, Toshiko Tomisawa, Indrawati Oey

**Affiliations:** 1 Department of Bioscience and Laboratory Medicine, Hirosaki University Graduate School of Health Sciences, Hirosaki 036-8564, Japan; nnaoki@hirosaki-u.ac.jp; 2 Faculty of Agriculture and Life Science, Hirosaki University, Hirosaki 036-8561, Japan; hayatosp@hirosaki-u.ac.jp; 3 Department of Nursing Sciences, Hirosaki University Graduate School of Health Sciences, Hirosaki 036-8564, Japan; tmtott@hirosaki-u.ac.jp; 4 Department of Food Science, University of Otago, Dunedin 9054, New Zealand; indrawati.oey@otago.ac.nz; 5 Riddet Institute, Palmerston North 4442, New Zealand

**Keywords:** blackcurrant extract, phytoestrogen, elastin, vascular remodeling, ovariectomized rat

## Abstract

Estrogen exerts cardioprotective effects in menopausal women. Phytoestrogens are plant-derived substances exhibiting estrogenic activity that could beneficially affect vascular health. We previously demonstrated that blackcurrant (*Ribes nigrum* L.) extract (BCE) treatment exerted beneficial effects on vascular health via phytoestrogenic activity in ovariectomized (OVX) rats, which are widely used as menopausal animal models. Here, we examined whether BCE treatment reduced elastin degradation and prevented pathological vascular remodeling in OVX rats fed a regular diet (OVX Control) or a 3% BCE-supplemented diet (OVX BCE), compared with sham surgery rats fed a regular diet (Sham) for 3 months. The results indicated a lower staining intensity of elastic fibers, greater elastin fragmentation, and higher α-smooth muscle actin protein expression in OVX Control rats than in OVX BCE and Sham rats. Pathological vascular remodeling was only observed in OVX Control rats. Additionally, we investigated matrix metalloproteinase (MMP)-12 mRNA expression levels to elucidate the mechanism underlying elastin degradation, revealing significantly upregulated MMP-12 mRNA expression in OVX Control rats compared with that in Sham and OVX BCE rats. Together, we identify BCE as exerting a vascular protective effect through reduced MMP-12 expression and vascular smooth muscle cell proliferation. To our knowledge, this is the first report indicating that BCE might protect against elastin degradation and pathological vascular remodeling during menopause.

## 1. Introduction

Postmenopausal women are known to be at markedly increased risk of cardiovascular disease (CVD) [1,2]. This phenomenon is presumed to be caused by estrogen deficiency in postmenopausal women. A previous animal study demonstrated that estrogen provides significant protection against atherosclerosis development [3]. Hormone-replacement therapy (HRT) with estrogen is the most effective treatment for menopausal symptoms in healthy women, but the risks and benefits associated with HRT remain uncertain. HRT is associated with a lower primary risk of CVD in postmenopausal women [4,5]; however, HRT has not shown a reduction in CVD events in primarily older postmenopausal women [1,6]. As health outcomes generally improve, in the future, it is conceivable that the percentage of primarily older postmenopausal women would increase. Thus, there is a need to develop safe alternatives to hormonal treatment.

Blackcurrants (*Ribes nigrum* L.) contain high levels of anthocyanins, including cyanidin-3-glucoside, cyanidin-3-rutinoside, delphinidin-3-glucoside, and delphinidin-3-rutinoside [7]. These anthocyanins have been reported to exert some health benefits, such as the prevention of breast cancer and reduction in inflammation and obesity [8,9,10]. Furthermore, bioactive compounds found in blackcurrants have been traditionally used to treat various conditions such as rheumatic disease. [11]. Phytoestrogens are plant compounds that have an estrogenic activity mediated by estrogen receptors (ERs) [12]. We had previously reported that blackcurrant anthocyanins have phytoestrogenic activity mediated via ERα [13] and ERβ [14]. Moreover, polyphenol-rich blackcurrant extract (BCE) has shown beneficial effects via phytoestrogenic activity, such as cosmetic improvement of the skin [15], alleviation of hair loss [16], improvement in vascular endothelium function [17], and alleviation of lipid metabolism abnormalities [18] in ovariectomized (OVX) rats.

Elastin, which is the dominant extracellular matrix (ECM) protein deposited in the arterial wall [19], plays an important role in determining the mechanical strength of vessels at low pressure [20]. Additionally, estrogen promotes an elastic matrix profile, which is likely to influence large artery stiffness [21]. Furthermore, vascular remodeling can lead to various pathological vascular disorders, such as hypertension, atherosclerosis, and lower-extremity venous disease [22,23,24,25,26]. Phytoestrogens are assumed to improve menopausal symptoms; however, their effects on vascular diseases such as atherosclerosis remain unclear [1].

Accordingly, the present study aimed to clarify the beneficial effects of BCE on vascular health. For this purpose, we used OVX rats as the menopausal animal model to examine whether BCE prevented elastin degradation and pathological vascular remodeling during menopause. To our knowledge, this is the first study to report the effects of the dietary intake of BCE on vascular health in OVX rats.

## 2. Materials and Methods

### 2.1. Blackcurrant Extract and BCE-Containing Feed

In this study, the powdered form of blackcurrant extract (CaNZac-35) was purchased from Koyo Mercantile Co. (Tokyo, Japan). This BCE powder contained high concentrations of anthocyanins (38.0 g/100 g BCE) [13]. BCE-containing feed was prepared by supplementing the AIN-93M diet with 3% BCE.

### 2.2. Animals and Treatments

This study was approved by the Animal Research Committee of Hirosaki University (permission number: G 18003) and was conducted in accordance with the guidelines for animal experimentation of Hirosaki University. Ovariectomy and sham surgery rat treatment methods were performed according to our previous study [17]. Briefly, 12-week-old, female, Sprague-Dawley rats were purchased from CLEA Japan, Inc. (Tokyo, Japan), divided into 3 groups, and fed a diet supplemented with and without 3% BCE (as indicated) for 3 months. The rat groups included (1) Sham: sham surgery rats without BCE treatment (*n* = 5), (2) OVX Control: OVX rats without BCE treatment (*n* = 6), and (3) OVX BCE: OVX rats treated with 3% BCE (*n* = 5). We used 3% BCE because this amount was previously shown to strongly exert phytoestrogenic effects in rats [13]. At the end of the experiment, the animals were euthanized and the abdominal aorta was removed.

### 2.3. Histological Analyses

Rat abdominal aortic tissue samples were subjected to Elastica van Gieson staining (Muto Pure Chemicals, Tokyo, Japan) for histological analysis and elastic fiber content was evaluated. Aortic tissues were cut into 3–4 specimens (200-μm thick), fixed in 10% formalin neutral buffer solution, and routinely processed for paraffin embedding. Serial 3-μm-thick sections were then cut and placed on glass slides for Elastica van Gieson staining. The stained specimens were then photographed using an AX80 DP21 digital microscope camera (Olympus, Tokyo, Japan) interfaced with a computer and evaluated.

Staining intensity scores of the elastic fibers were semi-quantitatively determined as follows: 1 (weak), 2 (moderate), and 3 (intense). Simultaneously, we semi-quantitatively counted the number of thick elastic fiber layers in the aortic tunica media. Additionally, the elastin break positivity rate was calculated as the percentage of elastin break-positive specimens among the total number of specimens. Furthermore, we evaluated the number of elastin breaks in the stained elastin break-positive sections.

### 2.4. Immunofluorescence Staining of α-Smooth Muscle Actin (α-SMA) Protein

For immunofluorescence staining, the tissues were deparaffinized and endogenous peroxidases in the specimens were blocked using Peroxidase-Blocking Solution (DakoCytomation A/S, Agilent Technologies, Inc., Santa Clara, CA, USA) at room temperature for 5 min. Before immunohistochemical staining, we performed an antigen retrieval step by boiling the specimens in 10 mM citrate buffer (pH 6.0) using a microwave oven for 20 min. After incubation with Protein Block Serum-Free Reagent (DakoCytomation A/S) at room temperature for 5 min, the specimens were incubated with rabbit anti-α-SMA polyclonal antibody (1:100; Proteintech Group, Chicago, IL, USA) at room temperature for 60 min, and then washed. The specimens were then incubated with anti-rabbit immunoglobulin/TRITC (1:40, DakoCytomation A/S) at room temperature for 30 min. Nuclear staining and mounting were performed using VECTASHIELD^®^ Mounting Medium with DAPI (Vector Laboratories, Burlingame, CA, USA). The specimens were observed under a BZ-X700 fluorescence microscope (KEYENCE, Tokyo, Japan), and α-SMA fluorescence intensity was measured as the average intensity per unit area along the aorta using BZ-X800 Analyzer version 1.1.1.8 software (KEYENCE, Tokyo, Japan).

### 2.5. Quantitative Reverse Transcription-Polymerase Chain Reaction (RT-qPCR)

Matrix metalloproteinase (*Mmp*)-9 and *Mmp-12* mRNA expression levels were evaluated by RT-qPCR analysis, as previously described [17]. Briefly, flash-frozen sections of the abdominal aorta were ground using a homogenizer, and total RNA was extracted using the RNeasy Mini Kit (Qiagen, Hilden, Germany). cDNA was reverse-transcribed from total RNA (200 ng) using the PrimeScript^®^ RT Master Mix (TaKaRa Bio Inc., Shiga, Japan). Specific mRNA levels were quantified by qPCR using SYBR^®^ Premix ExTaq™ II (TaKaRa Bio Inc.). The PCR amplification conditions were as follows: preheating at 95 °C for 10 min for initial denaturation, followed by 40 cycles at 95 °C for 15 s and 60 °C for 30 s. The transcript levels of target genes were normalized to that of glyceraldehyde 3-phosphate dehydrogenase (*Gapdh*) in rats. The primers used for qPCR were as follows: *Gapdh*, forward 5′-AGGCCGGTGCTGAGTATGTC-3′ and reverse 5′-TGCCTGCTTCACCACCTTCT-3′ [27]; *Mmp-9*, forward 5′-CTGCAGTGCCCTTGAACTAA-3′ and reverse 5′-TATCCGGCAAACTAGCTCCT-3′ [27]; and *Mmp-12*, forward 5′-GCTGGTTCGGTTGTTAGG-3′ and reverse 5′-GTAGTTACACCCTGAGCATAC-3′ [28]. PCR specificity was verified by melting curve analysis. All samples were analyzed in triplicate, and relative gene expression was calculated using the 2^−ΔΔCt^ method.

### 2.6. Statistical Analysis

All statistical analyses were performed using bell curve analysis with Excel software v3.10 (Social Survey Research Information, Tokyo, Japan). Normality was confirmed by the Shapiro-Wilk test; all data showed a non-normal distribution. Kruskal-Wallis analysis with the Steel-Dwass post-hoc test was performed for multiple comparisons between three groups, while the Mann-Whitney U test was used for comparison between two groups. A *p* value < 0.05 was considered statistically significant. Data are shown as the mean ± standard error of the mean (SEM) of at least three independent experiments.

## 3. Results and Discussion

### 3.1. Evaluation of Elastic Fibers in the Abdominal Aorta of OVX Rats Subjected to Dietary Intake of BCE

**Figure 1:**
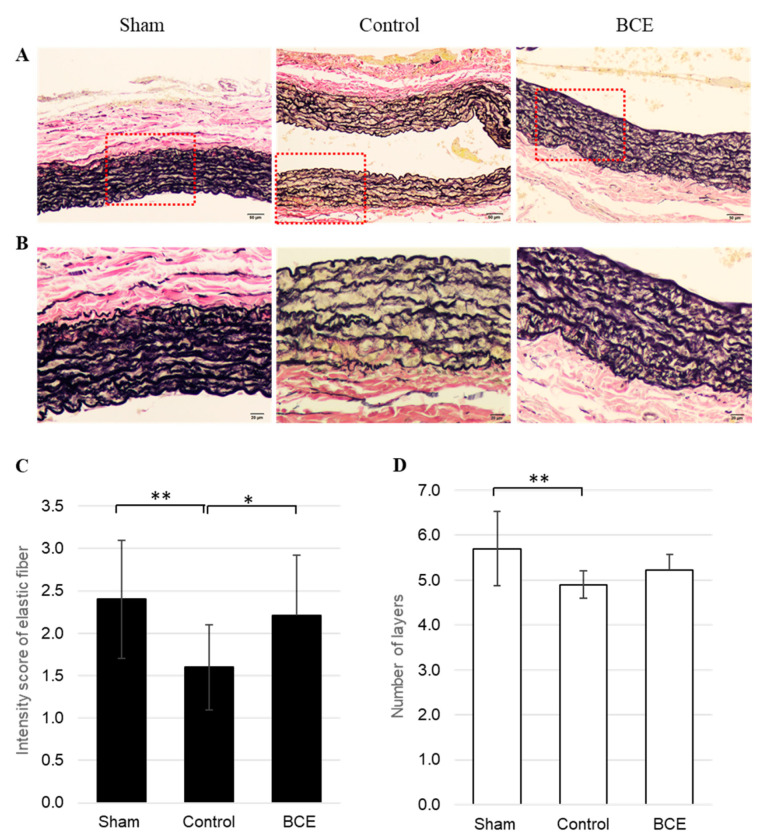
Representative images and semi-quantification of elastic fibers in Elastica van Gieson-stained tissues. (**A**) 100× magnification (scale bar = 50 µm) and (**B**) 200× magnification (scale bar = 20 µm) of the boxed area shown in (**A**). (**C**) Staining intensity of elastic fibers in the aortic tunica media semi-quantified at 3 intensities: 1, 2, and 3. (**D**) Evaluation of the number of elastic fiber layers in the aortic tunica media. Data are shown as means ± SEM; *n* = 10 (Sham), *n* = 20 (Control) and *n* = 14 (BCE). * *p* < 0.05, ** *p* < 0.01. Sham, sham surgery rats; Control, OVX rats without BCE treatment; BCE, OVX rats treated with 3% BCE; OVX, ovariectomized; BCE, blackcurrant (*Ribes nigrum* L.) extract; SEM, standard error of the mean.

We assessed the effects of BCE on elastic fibers in the abdominal aorta of OVX rats. Since OVX rats do not produce estrogen, they are considered the ideal animal model of menopause. Elastic fibers were identified in the abdominal aorta by Elastica van Gieson staining (Figure 1A,B). The staining intensity of elastic fibers was semi-quantitatively evaluated, revealing a decreased staining intensity score for the elastic fibers in OVX BCE and Control rats (2.2 ± 0.7 and 1.6 ± 0.5, respectively), compared to that in Sham rats (2.4 ± 0.7) (Figure 1C). The elastic fiber staining intensity score was significantly lower in OVX Control rats than in Sham rats (*p* < 0.01), but did not differ significantly between the Sham and OVX BCE rats. Furthermore, the elastic fiber staining intensity score was significantly higher in OVX BCE rats than in OVX Control rats (*p* < 0.05). Similarly, the number of elastic fiber layers in the aortic tunica media was significantly lower in OVX Control rats than in Sham rats (*p* < 0.01), but did not differ significantly between the Sham and OVX BCE rats (Figure 1D).

### 3.2. Evaluation of Elastin Breaks in the Abdominal Aorta of OVX Rats Subjected to Dietary Intake of BCE

**Figure 2:**
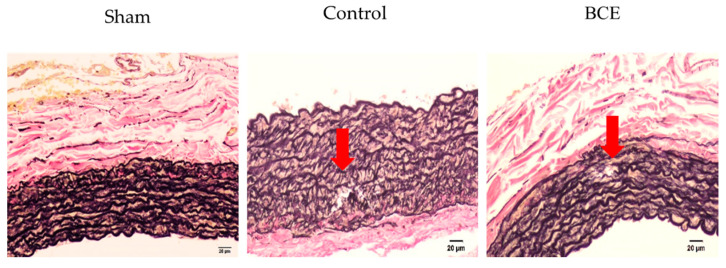
Representative images of elastin breaks in Elastica van Gieson stained tissues (200× magnification, scale bar = 20 µm). Arrows indicate elastin breaks. No elastin breaks were observed in Sham rats. Sham, sham surgery rats; Control, OVX rats without BCE treatment; BCE, OVX rats treated with 3% BCE; OVX, ovariectomized; BCE, blackcurrant (*Ribes nigrum* L.) extract.

Next, we investigated whether BCE treatment regulated elastin degradation in OVX rats. The elastin breaks were observed only in OVX Control and OVX BCE rats. No obvious elastin breaks were observed in the Sham rats (Figure 2).

Since no elastin breaks were observed in Sham rats, we evaluated elastin breaks in OVX Control rats and OVX BCE rats. The elastin break positivity rate was reduced in OVX BCE rats (21.4%) compared with that in OVX Control rats (35%) (Table 1).

Additionally, the number of elastin breaks was evaluated in the elastin break-positive sections of OVX Control and OVX BCE rats (Table 2).

The number of elastin brakes in each elastin brake positive sections was higher in OVX Control (1.3) than in OVX BCE rats (1.0); however, the difference was not statistically significant. This might be attributable to a lack of statistical power, since the number of elastin break-positive specimens was low.

Our results indicated that elastin levels were decreased in OVX Control rats, while BCE treatment maintained elastic fibers and prevented elastin fragmentation. These results were consistent with those of our previous study [15], in which elastin mRNA expression was significantly upregulated in anthocyanin- and BCE-treated human fibroblasts, compared with that in untreated control cells. Similarly, the elastin protein level was also increased in the cytoplasm of anthocyanin- and BCE-treated cell lines, as demonstrated by immunofluorescence staining. Moreover, the in vivo portion of this study revealed visibly less elastic fiber content in the skin tissue of OVX Control rats than in OVX BCE and Sham rats [15], which concurred with the results obtained in the present study. It is well known that estrogen plays a key role in maintaining the structural and functional integrity of the skin. It has been reported that estrogen is involved in the regulation of elastin metabolism in the skin [29,30,31]. However, to date, there has been limited research on the relationship between elastin and estrogen in blood vessels [21]. Only a few studies have demonstrated that exogenous estradiol improved arterial stiffness in OVX mice [32]. Our results suggested that the reduction in estrogen in OVX rats decreased elastin levels. Additionally, we previously reported that BCE exerted phytoestrogenic effects [13,14,15,16,17,18]; hence, we speculated that BCE treatment significantly alleviated the decrease in elastic fiber layers and increase in elastin fragmentation in OVX rats through phytoestrogenic activity.

### 3.3. α-SMA Protein Expression in the Abdominal Aorta of OVX Rats Subjected to Dietary Intake of BCE, as Evaluated by Immunofluorescence Staining

**Figure 3:**
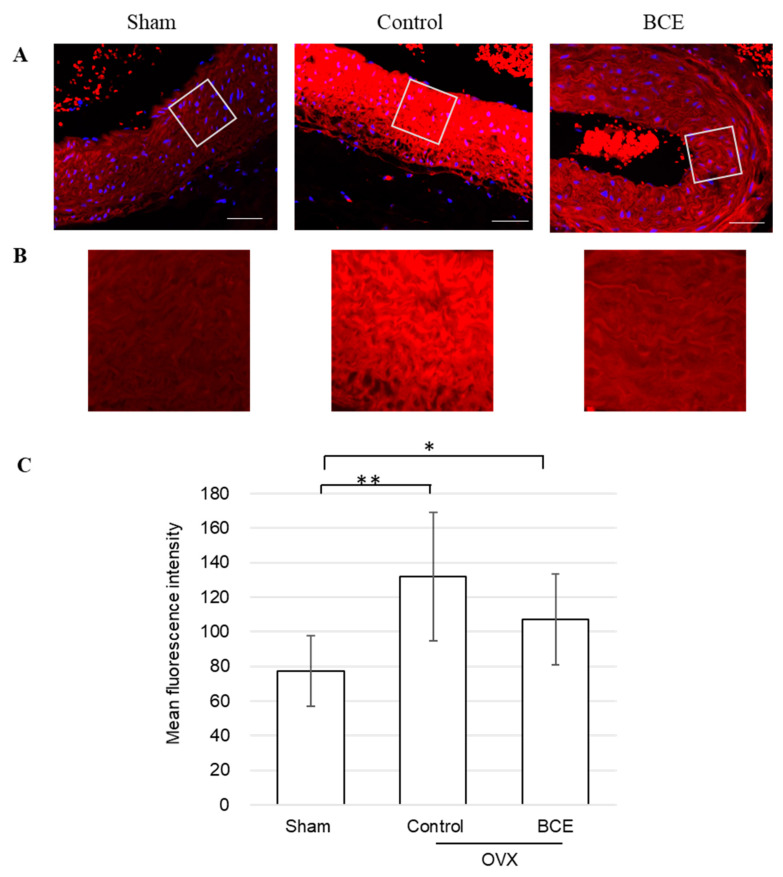
Representative images of immunofluorescence-stained tissues, evaluated for α-SMA protein expression. (**A**) Smooth muscle cells stained with TRITC (red) and counter-stained with DAPI (blue) to visualize the nuclei (400× magnification; scale bar = 50 µm). (**B**) Fluorescence intensity of the images enlarged for clarity. (**C**) Quantification of α-SMA protein fluorescence. Data are shown as means ± SEM; *n* = 11 (Sham), *n* = 12 (Control) and *n* = 11 (BCE). * *p* < 0.05, ** *p* < 0.01. OVX, ovariectomized; BCE, blackcurrant (*Ribes nigrum* L.) extract; SMA, smooth muscle actin; SEM, standard error of the mean.

Next, we assessed α-SMA protein expression in the abdominal aorta of OVX rats by immunofluorescence staining (Figure 3A,B). α-SMA is the actin isoform that predominates within vascular smooth muscle cells (VSMCs) [33]. α-SMA protein expression was significantly higher in both OVX Control and OVX BCE rats than in Sham rats (*p* < 0.01 and *p* < 0.05, respectively), corresponding with other studies in which estrogen inhibited VSMC proliferation [34,35,36]. Furthermore, α-SMA protein expression was decreased in OVX BCE rats compared with that in OVX Control rats (Figure 3C). These results were consistent with those of elastin fragmentation (Figure 2), but showed the opposite trend to elastic fiber staining intensity (Figure 1C) and number of elastic fiber layers (Figure 1D). At first, we speculated that BCE reduced α-SMA expression through phytoestrogenic activity, but α-SMA expression was significantly higher in OVX BCE rats than in Sham rats (*p* < 0.05). Thus, our results suggested that BCE might exert preventive effects on VSMC proliferation not only via phytoestrogenic activity, but also other pathways. Intact elastin has been reportedly associated with a contractile phenotype of VSMCs [37]. Other studies have also reported that elastin is a potent autocrine regulator of VSMC activity and stabilizes vascular structure by inducing a quiescent contractile state in VSMCs [19,38]. Our results indicated that α-SMA expression was decreased in OVX BCE rats compared with that in OVX Control rats, which correlated with increased elastic fiber numbers and decreased elastin degradation. The reduced α-SMA protein expression might have been caused by elastin fragmentation or reduction in elastic fiber abundance, rather than the phytoestrogenic activity of BCE.

### 3.4. Evaluation of Pathological Vascular Remodeling of the Abdominal Aorta in OVX Rats Subjected to Dietary Intake of BCE

**Figure 4:**
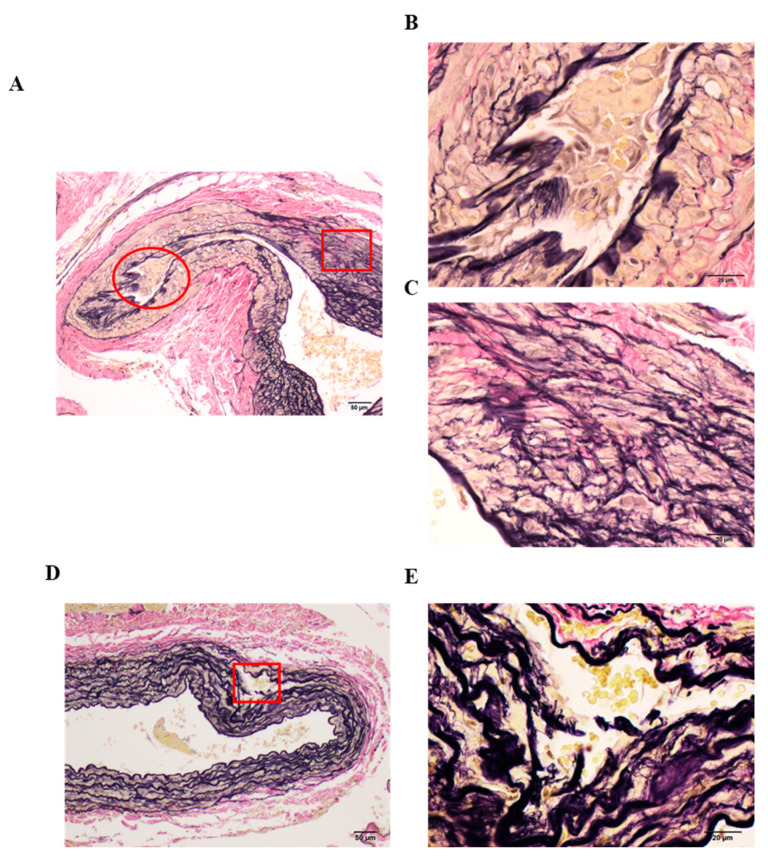
Representative images of pathological vascular remodeling in Elastica van Gieson-stained tissues of OVX Control rats. (**A**,**D**) Low magnification (100×, scale bar = 50 µm); (**B**,**C**,**E**) high magnification (400×, scale bar = 20 µm), magnification of the boxed areas in (**A**,**D**). (**B**) high magnification of the red circle area; (**C**) high magnification of the red boxed area in (**A**); (**E**) high magnification of the red boxed area in (**D**). OVX, ovariectomized.

We observed apparent pathological vascular remodeling only in some parts of OVX Control rats (Figure 4A). The smooth muscle cells (SMCs) (yellow) proliferated and migrated, replacing elastic fibers (black) in the aortic tunica media of OVX Control rats. Additionally, VSMC proliferation and migration caused vascular occlusion, which could be observed in Figure 4B. Furthermore, as shown in Figure 4C, elastic fibers (black) were notably decreased and collagen fibers (red) and SMCs (yellow) were increased. Additionally, we confirmed obvious elastin degradation, partial thickening of the blood vessel wall, and abnormal structures (Figure 4D). Figure 4E shows elastic fiber degradation, and entry of erythrocytes into the affected part. Our results revealed that pathological vascular remodeling occurred only in some of the specimens from OVX Control rats, whereas no remodeling was observed in Sham and OVX BCE rats, which had normal blood vessel structure (supplementary Figure S1).

The majority of VSMCs in blood vessels exhibit the contractile phenotype in the normal state [37]. However, in the state of vascular injury or inflammation, VSMCs switch from the contractile phenotype to the synthetic phenotype, thereby playing an important role in vascular remodeling [39,40,41]. Estrogen can effectively prevent this switch [42]. As previously described, estrogen inhibits many processes, including VSMC migration and proliferation, via genomic and non-genomic mechanisms during vascular remodeling [3,35,36]. Thus, our results suggested that the phytoestrogenic effects exerted by BCE effectively prevented vascular remodeling. Furthermore, arterial ECM remodeling can lead to arterial stiffening, which is thought to reflect changes in ECM protein synthesis and MMP-mediated ECM degradation [23]. MMPs induce structural changes in the vessel wall by rearranging collagen and elastin [37]. Therefore, we investigated *Mmp* mRNA expression to clarify the mechanism underlying elastin degradation and determine whether BCE treatment suppressed *Mmp* mRNA expression. MMP-12 is well known as a potent elastase [32,43], and MMP-9 has been reportedly implicated in elastin breakdown [44].

### 3.5. RT-qPCR Analysis of Mmp Levels in the Abdominal Aorta of OVX Rats Subjected to Dietary Intake of BCE

**Figure 5:**
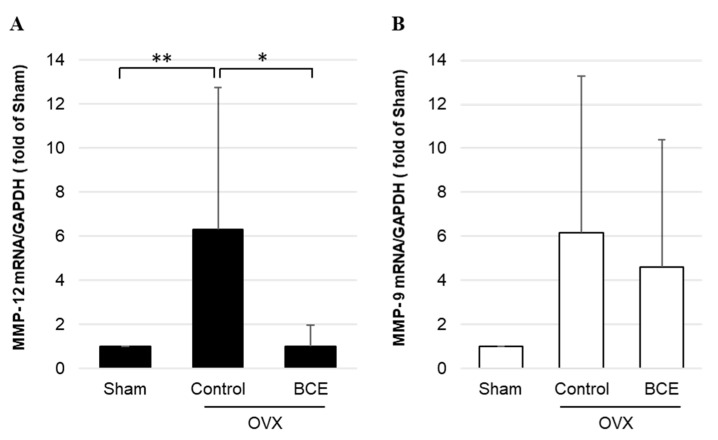
*Mmp* mRNA levels in OVX BCE rats. (**A**) *Mmp*-12 and (**B**) *Mmp*-9 mRNA levels quantified by RT-qPCR. Data are shown as the mean ± SEM of at least three independent experiments (*n* = 9). * *p* < 0.05, ** *p* < 0.01, vs. Sham rats. Sham, sham surgery rats; Control, OVX rats without BCE treatment; BCE, OVX rats treated with 3% BCE; OVX, ovariectomized; BCE, blackcurrant (*Ribes nigrum* L.) extract; MMP, matrix metalloproteinase; RT-qPCR, quantitative reverse-transcription polymerase chain reaction; SEM, standard error of the mean.

We investigated the effects of BCE on repression of *Mmp*-12 and *Mmp*-9 mRNA expression via RT-qPCR analysis (Figure 5). *Mmp*-12 mRNA levels were significantly upregulated in OVX Control rats compared with those in Sham rats, whereas no notable difference was observed between OVX BCE and Sham rats. Furthermore, *Mmp*-12 mRNA levels were significantly downregulated in OVX BCE rats compared with those in OVX Control rats (Figure 5A). Similarly, *Mmp*-9 mRNA levels were notably upregulated in both OVX Control and OVX BCE rats, compared with those in Sham rats. Additionally, *Mmp*-9 mRNA expression was downregulated in OVX BCE rats compared with that in OVX Control rats (Figure 5B).

These results corresponded with those of elastin fragmentation (Figure 2), suggesting that BCE might regulate elastin degradation via *Mmp*-12 expression. Our previous study reported that *Mmp*-12 mRNA levels were significantly decreased in BCE-treated human fibroblasts compared with those in untreated control cells [15], which concurred with the results of the present study. Another study determined that estrogen downregulated the *MMP*-12 expression in human and model rat macrophages [32]. Based on our results, we speculated that BCE might downregulate *MMP*-12 expression via phytoestrogenic activity. Furthermore, previous studies reported that MMP-12 was induced in SMCs in response to various pro-inflammatory stimuli; MMP-12 was induced in arterial VSMCs after acute vascular injury [23] and in human airway SMCs of patients with asthma, chronic obstructive pulmonary disease, and chronic cough [45]. Thus, our results suggested that the decrease in MMP-12 expression in OVX BCE rats might be due to the synergistic phytoestrogenic and preventive effects of BCE treatment on VSMC proliferation. Our results also indicated that BCE treatment possibly downregulated not only MMP-12, but also MMP-9. Several studies revealed that MMP-9 overexpression was associated with arteriosclerosis [46,47,48].

To date, upstream regulators of MMP-12 remain largely unknown [49]. Tissue inhibitors of metalloproteinase (TIMP) is the major cellular inhibitor of MMP [50]. Estrogen is known to be involved in the maintenance of TIMP-MMP balance and degradation of collagen in OVX rats [51]. Additionally, TIMP-3 is downregulated in metabolic and inflammatory disorders such as type 2 diabetes mellitus and atherosclerosis [52,53]. Thus, we investigated the effects of BCE on *Timp*-3 mRNA expression and found that *Timp*-3 mRNA expression was not significantly altered (supplementary Figure S2). However, an upward trend was observed in OVX BCE rats, compared with that in OVX Control rats. Furthermore, the fold change in *Timp*-3 mRNA expression in OVX BCE rats was similar to that in Sham rats; thus, we speculated that T*imp*-3 mRNA expression in OVX BCE was not affected by phytoestrogen treatment. These results were consistent with those of our previous study that showed that *TIMP*-3 mRNA expression in human skin fibroblasts was notably increased with BCE treatment compared with estrogen and anthocyanin treatment [15]. Thus, increased T*IMP*-3 mRNA levels might be implicated in other effects induced by BCE treatment, in addition to the phytoestrogenic effect.

Our results indicated that dietary intake of BCE effectively prevented vascular remodeling by suppressing VSMC proliferation and reducing elastin degradation by downregulating MMP-12 expression. Vascular remodeling has attracted attention in relation to many vascular diseases, such as hypertension and arteriosclerosis [23,24,25,26]. Additionally, since MMP-12 activation increases elastin degradation and large artery stiffness [49], it may be critical for the initiation and progression of atherosclerosis [54]. We previously reported the beneficial effects of BCE on vascular health. BCE strongly increased endothelial nitric oxide synthase (*eNOS*) mRNA expression and nitric oxide production in human endothelial cells, and dietary BCE increased *eNOS* protein expression in an OVX rat model [17]. Additionally, BCE effectively prevented lipid-associated metabolic abnormalities [18] and attenuated smoking-induced acute endothelial dysfunction and improved peripheral temperature in young smokers [55]. Furthermore, the amount of BCE administered in the animal model in the current study was equivalent to the daily dose of polyphenols (1.9 g/60 kg body weight) provided by BCE (5.1 g) previously administered to humans [18]. This intake of polyphenols is considered realistic, and it has been speculated that continuous intake of BCE improves blood vessel health.

Additionally, several studies have reported the relationship between atherosclerosis and intake of various food components. The use of an isoflavonoid-rich herbal preparation in postmenopausal women may suppress the formation of new atherosclerotic lesions [1]. The antioxidant properties of red wine resveratrol are known to provide protection against coronary heart disease [56]. Further, dietary sea cucumber can potentially eliminate atherosclerosis [57]. However, few studies have reported the potential effects of BCE in preventing vascular disorders. BCE may function via the activity of several phytochemicals in menopausal vascular remodeling, including phytoestrogen; thus, further studies are warranted to completely elucidate the mechanisms underlying BCE activity.

## 4. Conclusions

To the best of our knowledge, this is the first report demonstrating that BCE intake effectively prevented elastin degradation and vascular remodeling in menopausal model rats. The present study indicated that dietary BCE prevents elastin degradation by downregulating *Mmp-12* mRNA expression and suppresses VSMC proliferation in OVX rats. Our results suggest that BCE intake might exert beneficial health effects on blood vessels in postmenopausal women. In this study, we did not administer BCE to humans; however, since the prevention of elastin degradation and pathological vascular remodeling is critical for maintaining vascular integrity, we intend to perform clinical studies in the future.

## Figures and Tables

**Table 1:** Elastin break positivity rate expressed as a percentage.

	Sham	OVX Control	OVX BCE
Number of elastin break-positive specimens	0	7	3
Total number of specimens	10	20	14
Number of elastin break-positive specimens/total number of specimens (%)	0	35.0	21.4

OVX, ovariectomized; BCE, blackcurrant (*Ribes nigrum* L.) extract.

**Table 2:** Semi quantification in Number of elastin brakes per Elastin brake positive sections.

	OVX Control	OVX BCE
Number of elastin brakes	9	3
Elastin brake positive sections	7	3
Number of elastin brakes/Elastin positive sections	1.3	1.0
SD	0.5	0

OVX, ovariectomized; BCE, blackcurrant (*Ribes nigrum* L.) extract; SD, Standard deviation.

## Data Availability

The data presented in this study are available in the article.

## Supplementary Materials

The following is available online at https://www.mdpi.com/2072-6643/13/2/560/s1, Figure S1: Representative images of structurally normal vessels at Elastica van Gieson stain in Sham and BCE rats. Figure S2: TIMP3 mRNA expression in BCE-treated OVX rats quantified by RT-qPCR.

## Abbreviations

α-SMA	alpha-smooth muscle actin
BCE	blackcurrant (*Ribes nigrum* L.) extract
CVD	cardiovascular disease
ECM	extracellular matrix
ER	estrogen receptor
GAPDH	glyceraldehyde 3-phosphate dehydrogenase
HRT	Hormone-replacement therapy
MMP	matrix metalloproteinase
OVX	ovariectomized
SEM	standard error of the mean
SMCs	smooth muscle cells
TIMP	tissue inhibitor of metalloproteinase
VSMCs	vascular smooth muscle cells
