# Supplementary material for: Blackcurrant (Ribes nigrum L.) Extract Exerts Potential Vasculoprotective Effects in Ovariectomized Rats, Including Prevention of Elastin Degradation and Pathological Vascular Remodeling
Source: Nutrients. 2021 Feb 8;13(2):560. doi: 10.3390/nu13020560 (PMC7915542; doi:10.3390/nu13020560)
Supplement: Supplementary file 1 [file nutrients-13-00560-s001.pdf]

Supplementary Files

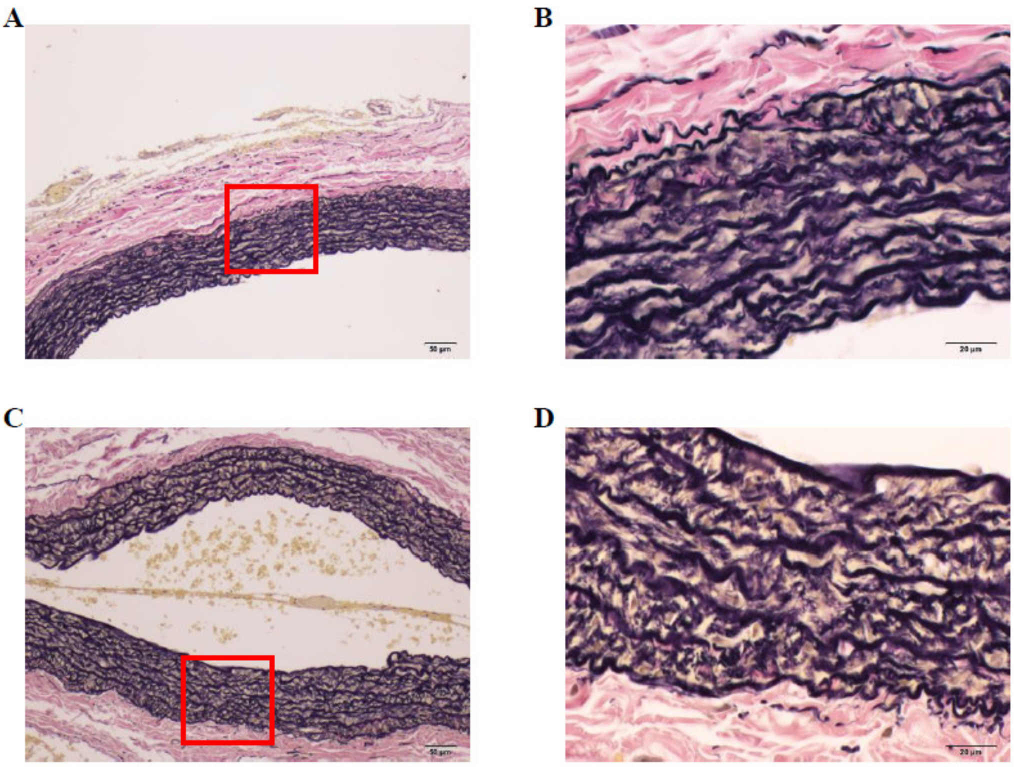

Supplementary Figure S1. Representative images of structurally normal vessels at Elasticavan Giesonstain in Sham (**A**) and BCE (**C**) rats, lower magnification (×100, scale bar = 50 μm). (**B,D**) were higher magnification boxed area shown in panel A and C (magnification ×400, scale bar = 20 μm).

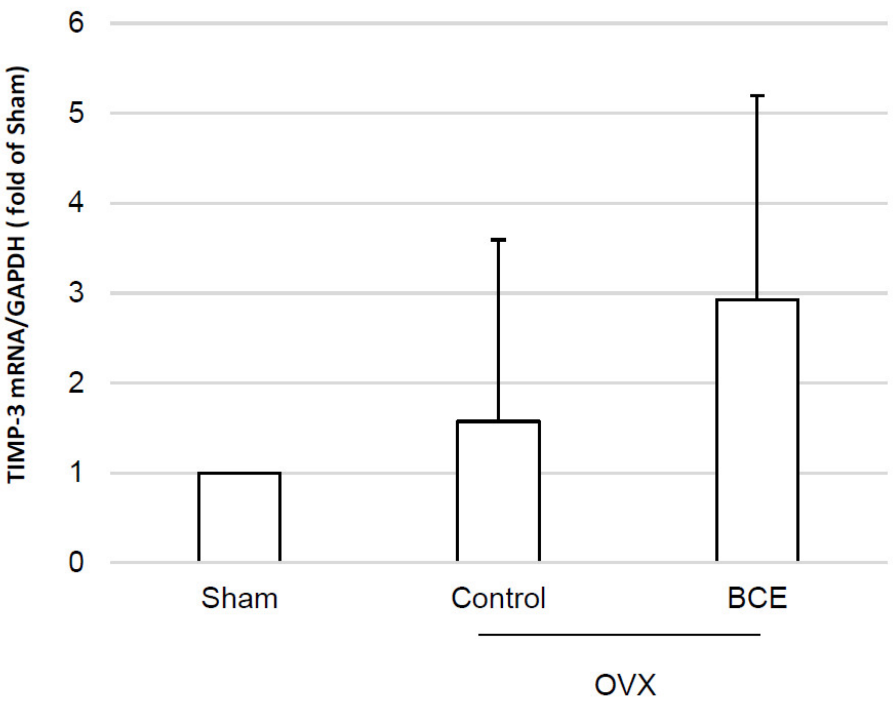

Supplementary Figure S2. TIMP3 mRNA expression in BCE-treated OVX rats quantified by RT-qPCR. Data are shown as the mean ± standard error of the mean of at least three independent experiments. .
